# Battery Charge Curve Prediction via Feature Extraction and Supervised Machine Learning

**DOI:** 10.1002/advs.202301737

**Published:** 2023-07-02

**Authors:** Laisuo Su, Shuyan Zhang, Alan J. H. McGaughey, B. Reeja‐Jayan, Arumugam Manthiram

**Affiliations:** ^1^ Materials Science and Engineering Program & Texas Materials Institute The University of Texas at Austin Austin TX 78712‐1591 USA; ^2^ Department of Mechanical Engineering Carnegie Mellon University Pittsburgh PA 15213 USA

**Keywords:** batteries, charge curves, feature extraction, prediction, machine learning

## Abstract

Real‐time onboard state monitoring and estimation of a battery over its lifetime is indispensable for the safe and durable operation of battery‐powered devices. In this study, a methodology to predict the entire constant‐current cycling curve with limited input information that can be collected in a short period of time is developed. A total of 10 066 charge curves of LiNiO_2_‐based batteries at a constant C‐rate are collected. With the combination of a feature extraction step and a multiple linear regression step, the method can accurately predict an entire battery charge curve with an error of < 2% using only 10% of the charge curve as the input information. The method is further validated across other battery chemistries (LiCoO_2_‐based) using open‐access datasets. The prediction error of the charge curves for the LiCoO_2_‐based battery is around 2% with only 5% of the charge curve as the input information, indicating the generalization of the developed methodology for predicting battery cycling curves. The developed method paves the way for fast onboard health status monitoring and estimation for batteries during practical applications.

## Graphical abstract

A methodology to accurately predict the entire battery charge curve with limited information that can be collected in a short period of time is presented, paving the way for fast onboard battery state estimation and health status monitoring during practical applications.

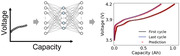

## 1 Introduction

Lithium‐ion batteries (LIBs) are becoming the dominant rechargeable batteries and are widely used in portable electronic devices and electric vehicles (EVs).^[^ ^1^, ^2^, ^3^ ^]^ Hundreds or even thousands of LIBs are connected to provide sufficient energy for EVs. For example, the Standard‐range version of the Tesla Model 3 carries 2,976 LIBs arranged in 96 groups of 31 cells and the Long‐range version contains 4,416 LIBs arranged in 96 groups of 46 cells.^[^ ^4^ ^]^ The failure of one battery could propagate quickly through the entire battery pack, which triggers the malfunction of the battery system and may lead to safety issues like smoke, fire, and explosion.^[^ ^5^ ^]^ Therefore, the states, such as state of charge (SOC) and remaining energy, and statuses, such as health condition, of batteries need to be accurately monitored to ensure their reliable and safe use.

A battery management system (BMS) is generally adopted to monitor the state of batteries, record battery usage information, analyze the status of batteries, and provide feedback and suggestions to customers.^[^ ^6^ ^]^ The BMS can directly measure some key information with sensors, such as voltage, current, and temperature.^[^ ^7^ ^]^ The combination of this information can further estimate the state of each battery, including SOC, remaining energy, and health conditions. Accurately estimating the health conditions of LIBs is very important, but challenging, to guide the use of batteries and at the same time prevent accidents and malfunctions.^[^ ^8^ ^]^ The health condition of a battery is generally reflected by a decreased maximum capacity, growth of internal resistance, and appearance of fatal aging mechanisms, such as formation of lithium dendrite.^[^ ^9^ ^]^ The assessment of these parameters is not trivial as BMSs typically only sample charging/discharging current and voltage of batteries at a SOC range that is defined by the usage habits of customers.^[^ ^10^ ^]^

Many efforts have been made to estimate the health state of batteries in real applications. One common method is based on models, such as equivalent circuit models^[^ ^11^ ^]^ and mechanism‐based models,^[^ ^12^ ^]^ to simulate the behaviors of batteries, followed by various optimization algorithms and observations to identify the parameters in the models and the health states.^[^ ^13^ ^]^ The estimation capability of battery health states relies on the accuracy of the models and the optimization algorithms. Therefore, building a representative model is crucial. Data‐driven methods are gaining increasing attention for battery health estimation and prediction due to their flexibility.^[^ ^14^ ^]^ The data‐driven methods have been demonstrated to predict the state of health (SOH) and remaining useful life of LIBs using impedance spectroscopy^[^ ^15^ ^]^ and to predict the cycle life using information from early cycles.^[^ ^14^ ^]^

Various useful battery characteristic information can be derived from the charge curve, such as maximum capacity that can be used to calculate SOH, available battery capacity that can be used to estimate SOC, and other energy‐related states. For example, Feng et al. proposed a support vector machine‐based algorithm that can use a partial charging segment (15 min charging) to predict the battery SOH with less than 2% error for 80% of all the cases.^[^ ^16^ ^]^ Zheng et al. proposed an online capacity estimation method based on a partial charge curve that can be utilized for battery lifetime prediction.^[^ ^17^ ^]^ Duan et al. developed a convolutional neural network to accurately predict the battery impedance spectroscopy based on limited constant‐current charging information (500 mV voltage window).^[^ ^18^ ^]^ Recently, Tian et al. further applied a deep neural network to predict the complete charge curve of a battery based on a small portion of the curve, which can be further used to estimate the SOC and SOH of the battery.^[^ ^10^ ^]^ Therefore, the charge curve is important for understanding the status of a battery.

The charge curve of a battery depends on the chemistry of battery electrodes, the charging current, and the health status of the battery. As the first two parameters are known and measurable in real applications, quantifying the aging mechanisms, i.e., health status, of the battery is crucial for accurately predicting the charge curve. In this study, we compare the ability of human experts and machine learning algorithms to quantify the aging mechanisms of batteries. Unsupervised learning algorithms perform better in capturing features necessary to quantify the battery aging mechanisms, which agrees well with existing studies that show the advantages of machine learning algorithms for feature extraction and selection.^[^ ^19^, ^20^ ^]^ These features were further used as the inputs for predicting the complete charge curves. Different from the previous study that can only take one continuous charge curve segment as the input,^[^ ^10^ ^]^ our developed methodology can also take multiple separated segments as the input, which will largely increase the practical applicability of the method. Finally, we demonstrate the applicability of the developed methodology in predicting open‐source battery charge curves with different battery chemistries.

## 2 Experimental Section

### 2.1 Data Generation

Two datasets were used in this study, including a lab‐generated dataset and an open‐access dataset. The lab‐generated dataset was collected in CR‐2032 type coin cells with LiNiO_2_ as the cathode and Li metal as the anode.^[^ ^21^, ^22^, ^23^ ^]^ The coin cells were tested at a C/10 rate three times after assembling, followed by cycling at room temperature with a C/2 charge rate and 1C discharge rate. The C/2 charge curves of these cells were used for this study, and a total of 10066 charge curves were selected with a minimum charge capacity of 160 mA h g⁻^1^.

The open‐access dataset was also used to evaluate the developed methodology. A total number of 4522 charge curves were taken from the Center for Advanced Life Cycling Engineering (CALCE) dataset (CS2_3, CS2_8, CS2_9, CS2_21, CS2_33∼CS2_38) provided by the A. James Clark School of Engineering at the University of Maryland.^[^ ^24^ ^]^ The CALCE dataset was obtained from batteries with LiCoO_2_ as the cathode material with trace elements of manganese, which is different from the LiNiO_2_ cathode tested in our lab.

### 2.2 Feature Extraction

Charge curves were selected for this study because the charging protocols are more controllable than discharge protocols to provide more consistent input in real‐world applications. Three commonly used unsupervised learning algorithms were applied to extract features from the charge curves, which are principal component analysis (PCA), non‐negative matrix factorization (NMF), and Autoencoder (AE). PCA and NMF are techniques that can decompose a matrix **Q** into two separated matrices **W** and **H**, such that it can be written as Equation (1),

(1) $$Q_{i\mu }\approx \;{\left(WH\right)}_{i\mu }=\sum_{a=1}^{p}W_{ia}H_{a\mu }\;$$

where Q is an *n* × *m* matrix that contains all the raw data information. **W** and **H** have dimensions of *n* × *p* and *p* × *m*. The hyperparameter *p* is the number of features. The *p* columns of **W** can be interpreted as the features of charge curves, which will be used to predict the complete charge curves by a supervised learning model. *p* is chosen based on the prediction accuracy of the validation set for all feature extraction algorithms. Each column of **H** contains the weights in a one‐to‐one correspondence with a basis feature in **W**.^[^ ^25^ ^]^

To obtain the elements of **W** and **H**, an optimization problem with the objective function **Q** − **WH**_*F*_ was solved, where · _*F*_ is the Frobenius norm. The difference between PCA and NMF lay in the constraints on the optimization. In PCA, the columns of **W** were orthonormal, and the rows of **H** were orthogonal, such that a unique solution was guaranteed.^[^ ^26^ ^]^ In NMF, the elements of **Q**, **W,** and **H** were constrained to be non‐negative. There is no unique solution because the problem is non‐convex.^[^ ^27^ ^]^ As such, we employed an initialization scheme called non‐negative double singular value decomposition, which rapidly reduces the approximation error to a value that is lower than that using a random initialization.^[^ ^28^ ^]^ PCA and NMF are performed using the Scikit‐Learn package.^[^ ^29^ ^]^

Autoencoder is an unsupervised learning method that adopts neural network architectures for the task of feature learning.^[^ ^30^ ^]^ The neural network is constructed with a bottleneck layer that enforces a compressed representation of the input layer, which has the same dimension as the output layer. The autoencoder with a single hidden layer implemented in this work is shown in Figure S1 (Supporting Information). This autoencoder is similar to NMF in that the hidden layer contains the weight matrix (**H**) corresponding to the charge curves in the decoder weight matrix (**W**),^[^ ^31^ ^]^ and the product of the two matrices approximates the input charge curve matrix. The autoencoder differs from NMF that there is no non‐negativity constraint on the decoder weight matrix. It can also be easily extended by adding more fully connected layers or other layer types, such as recurrent neural networks and convolutional neural networks.^[^ ^31^ ^]^ The autoencoders are implemented using the Pytorch package.^[^ ^32^ ^]^

### 2.3 Charge Curve Prediction

**Figure 1:**
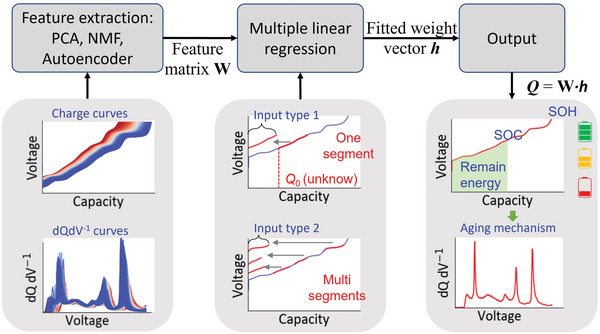
The workflow for predicting an entire charge curve of a battery based on a portion of the charge curve. Both a continuous segment and multiple separated segments can be used as the input. The output charge curve can derive many key states (SOC, SOH, and remaining energy) and even the aging mechanism of the battery using the dQ dV^−1^ analysis.

**Figure** **1** shows the workflow of this study. The 10066 charge curves of the LiNiO_2_ cells were randomly divided into a training set and a testing set with a ratio of 8 : 2, and 20% of the training set was used as the validation set to determine the hyper‐parameters in the models. Five‐fold cross‐validation was conducted to avoid overfitting. Features were extracted from the training dataset using PCA, NMF, and AE, from which we obtained the matrix **W** that contains *p* columns. Each column in matrix **W** represents a feature extracted from the training set.

#### 2.3.1 Single Segment Input

Given a partial charge curve with an arbitrary starting voltage that corresponds to a starting capacity *Q*_0_ and a voltage window length that corresponds to a capacity range of *Q_partial_*, it is assumed that it can be approximated by the linear combinations of the features in **W**. Thus, for the *i* ^th^ point on the partial charge curve, we can write the following Equation (2),

(2) $$Q_{0}+\Delta \;Q_{i}=\sum_{j=1}^{p}W_{j}\left(V_{i}\right)\cdot h_{j}\;+\varepsilon_{i}$$

where *Q*_0_ is the unknown starting capacity and is equivalent to the intercept of the linear regression and Δ*Q_i_* is the incremental capacity relative to the starting capacity *Q*_0_, obtainable from experimental measurements. **W**_*j*_ is the *j* ^th^ column of **W**, *h_j_* is the unknown weight corresponding to the features in **W**, and *ε* is the error that follows a normal distribution.

The relationship between the incremental capacity Δ*Q_i_* and the feature at a specific voltage **W**_*j*_(*V_i_*) can thus be modeled as a multiple linear regression problem. We use the *L_1_*‐norm as the regularization term to penalize the parameters and reduce overfitting. This regularizer (also called Lasso regularizer) can lead to some parameters being zero, i.e., removing the parameters for output evaluation.^[^ ^33^ ^]^ Thus, the Lasso regularizer can also serve as a feature selection method. Given a single‐segment partial charge curve with *n* points, the cost function can be defined as Equation (3),

(3) $$L\;\left(h,Q_{0}\right)=\sum_{i=0}^{n-1}{\left(Q_{0}+\Delta Q_{i}-\sum_{j=1}^{p}W_{j}\left(V_{i}\right)\cdot h_{j}\right)}^{2}\;+\lambda \sum_{j\;=\;1}^{p}\left|h_{j}\right|$$

where *λ* ≥ 0 is the regularization parameter that controls the trade‐off between approximation error and regularization strength.^[^ ^33^ ^]^ The hyperparameter *λ* is determined through the validation set.

Solving this multiple linear regression will lead us to the ***h*** and *Q*_0_ that minimize the cost function. To determine the complete charge curve *Q_complete_*, we just need to take the linear combination of the charge curves **W** obtained from feature extraction and the weight vector *h* corresponding to the partial charge curve,

(4) $$Q_{complete}=\;W\cdot h$$

The accuracy of the prediction is quantified by the root‐mean‐squared error (RMSE) between the predicted complete charge curve *Q_complete_* and the ground true charge curve *Q_true_*, as shown in Equation (5).

(5) $$\mathrm{RMSE}\;=\sqrt{\frac{1}{n}\sum_{i=0}^{n-1}{\left(Q_{complete}\left(i\right)-Q_{true}\left(i\right)\right)}^{2}}\;$$

#### 2.3.2 Multiple Separated Segments Input

In practical applications, regenerative braking was widely adopted in electric or hybrid vehicles to restore the wasted energy from the process of slowing down a car and using it to recharge the batteries.^[^ ^34^ ^]^ The process of regenerative braking results in many separated charge segments instead of a continuous charge curve during the charging process. These separated segments can also be used as the input in the model to predict the entire charge curve.

Given *m* input segments with *n* points in total, we have *m* starting capacity *Q*_0,*k*_ (*k* =  1, 2, …, *m*). This makes the multiple linear regression problem challenging to solve. For each segment, the linear relation at each point is displayed in Equation (6).

(6) $$Q_{0,k}+\Delta \;Q_{i,k}=\sum_{j=1}^{p}W_{j}\left(V_{i,k}\right)\cdot h_{j}\;+\varepsilon_{i,k}$$

All input segments share the same ***h*** but have different starting capacity *Q*_0,*k*_. This implies that the problem becomes *m* linear regressions with the same hyperplanes and different intercepts. We took the derivative of the input segments with respect to voltage, which generates the incremental capacity (IC) curves or dQ dV^−1^ curves. The dQ dV^−1^ analysis removes the unknown *Q*_0,*k*_ because the analysis is based on the relative change of capacity. For this approach to work, we need to create another dataset with dQ dV^−1^ curves and perform feature extraction to obtain the dQ dV^−1^ curve matrix **W**_*IC*_. The *i* ^th^ point on the *n* total points of the input can then be written as Equation (7),

(7) $${\left(\frac{dQ}{dV}\right)}_{i}=\sum_{j=1}^{p}W_{IC,j}\left(V_{i}\right)\cdot h_{IC,j}\;+\varepsilon_{IC,i}$$

where **W**_*IC*,*j*_ is the *j*th column of **W**_*IC*_. The cost function can be defined as Equation (8).

(8) $$L_{IC}\;\left(h_{IC}\right)=\sum_{i=0}^{n-1}{\left({\left(\frac{dQ}{dV}\right)}_{i}-\sum_{j=1}^{p}W_{IC,j}\left(V_{i}\right)\cdot h_{IC,j}\right)}^{2}+\lambda \sum_{j=1}^{p}\left|h_{IC,j}\right|\;$$

The complete dQ dV^−1^ curve is obtained by ${\left(\frac{dQ}{dV}\right)}_{compleate}=W_{IC}\;h_{IC}$, and the complete charge curve was recovered by integrating the dQ dV^−1^ curve with respect to the voltage.

## 3 Results and Discussion

### 3.1 Charge Curve Feature Extraction by Experts

**Figure 2:**
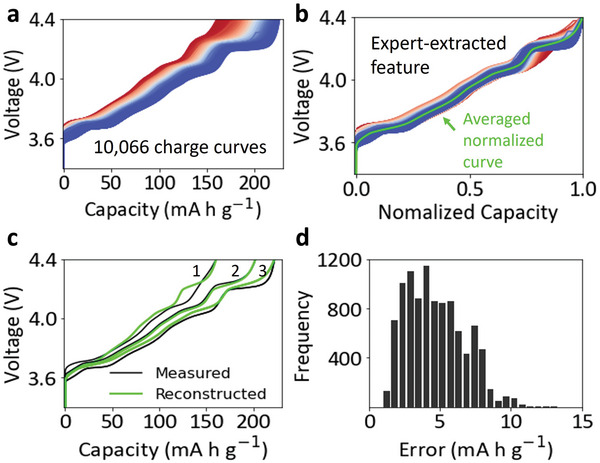
Battery charge curves feature extraction and reconstruction. a) The visualization of all the 10066 charge curves. b) Normalized charge curves and the averaged normalized charge curve calculated by taking the average of the normalized capacities at different voltages. c) Comparison between three measured charge curves and the corresponding reconstructed charge curves. d) Distribution of the reconstruction error of all the charge curves.

**Figure** **2**a displays all the charge curves collected in our lab with LiNiO_2_ as the cathode material, and the capacity distribution of these curves is shown in Figure S2 (Supporting Information). Figure 2b shows the normalized charge curves and the averaged normalized curve. A normalized charge curve was calculated by dividing its capacity by the maximum charge capacity of the curve. And the average normalized curve was calculated by taking the average of the normalized capacity for all the normalized curves at different voltages. This averaged normalized curve is considered an expert‐extracted feature, where the maximum charge capacity can be correlated to the amount of LiNiO_2_ active materials.

To quantify the capability of representing the battery degradation using the expert‐extracted feature (averaged normalized curve in Figure 2b), we compared the difference between the reconstructed charge curves and three tested charge curves (Figure 2c). The three charge curves were selected to be the ones with the maximum capacity (curve 3), the minimum capacity (curve 1), and the median capacity (curve 2). The reconstructed curve 2 matches well with the measured curve, while there are noticeable differences between the reconstructed and the measured data for curve 1 and curve 3. Moreover, Figure 2d summarizes the distribution of the reconstruction errors of all the charge curves calculated from Equation (9),

(9) $$Error\;=\sqrt{\frac{1}{n}\sum_{i=0}^{n-1}{\left(Q_{measured}\left(i\right)-Q_{reconstructed}\left(i\right)\right)}^{2}}\;$$

where *Q_measured_* (*i*) is the actual capacity value at the sampling point *i*, *Q_reconstructed_* (*i*) is the reconstructed capacity value at the sampling point *i*, and n is the total number of sampling points. The average error is 4.7 mA h g⁻^1^ with a standard deviation of 2.0 mA h g⁻^1^.

The averaged normalized curve in Figure 2b represents the characteristic voltage profile of LiNiO_2_ during delithiation, while the maximum charge capacity is determined by the amount of LiNiO_2_ active materials. If the loss of active materials is the only degradation mechanism for the capacity fading of LiNiO_2_ cells, all the charge curves can then be accurately reconstructed using the extracted feature (the averaged normalized curve) and the corresponding parameter (maximum charge capacity). However, there are many other degradation mechanisms, such as impedance growth and reaction heterogeneity.^[^ ^35^ ^]^ The impedance growth leads to an increased voltage polarization, which shifts the charge curve upward. Moreover, the impedance of a battery is a function of the SOC,^[^ ^36^ ^]^ complicating the reconstruction process of the charge curve. Similarly, the LiNiO_2_ electrode is composed of many secondary particles with a diameter of around 12 µm, which is further composed of hundreds of primary particles with a length of around 100 nm.^[^ ^21^ ^]^ The extraction of Li^+^ from these primary particles and secondary particles is nonuniform. The heterogeneity of the Li^+^ extraction also depends on the SOC and different aging status, making the reconstruction process of the actual charge curve even more complicated. Therefore, the features that represent other degradation mechanisms of LiNiO_2_ cells need to be considered and extracted to predict the health status of batteries.

### 3.2 Charge Curve Feature Extraction by Machine Learning

**Figure 3:**
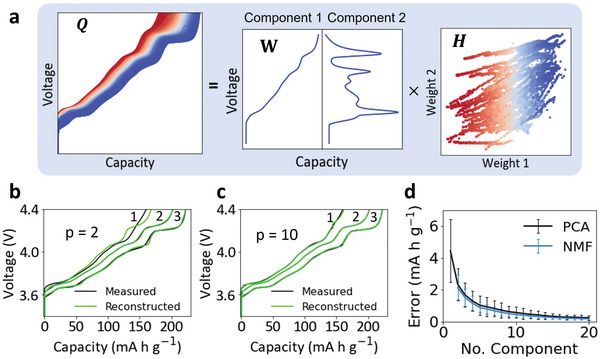
Battery charge curve feature extraction and reconstruction using unsupervised learning algorithms. a) Visual representation of charge curves matrix decomposition with two components. b,c) Comparison between three measured charge curves and the corresponding reconstructed charge curves based on PCA‐captured features with b) two components and c) ten components. d) Evolution of the reconstruction errors of all the 10066 charge curves with the number of components in the PCA and NMF. The error bar represents the standard deviation of the errors.

Unsupervised learning algorithms were further applied to extract features from the charge curves. **Figure** **3**a shows the decomposition of all the 10066 charge curves (Q) into two components (*p* = 2) and the corresponding weights using PCA. The mathematical principle of charge curve matrix decomposition can be found in Experimental Section. Component 1 has a similar shape as the expert‐extracted feature shown in Figure 2b, indicating that it represents the characteristic voltage profile of LiNiO_2_ during de‐lithiation and the corresponding weight 1 represents the amount of active materials. Interestingly, component 2 shows a similar shape to the dQ dV^−1^ analysis of the charge curve, where each phase transition corresponds to a peak in the dQ dV^−1^ curve.^[^ ^37^ ^]^ As Li^+^ diffusion kinetics within LiNiO_2_ cathode particles follows the same trend of the dQ dV^−1^ curve,^[^ ^23^ ^]^ component 2 may represent the kinetic effect of LiNiO_2_ during de‐lithiation, and the corresponding weight 2 represents the kinetic contribution to the overall charge capacity.

The accuracy of the reconstructed charge curve can be improved by increasing the number of components in the unsupervised learning algorithms. Figure 3b,c shows the reconstruction of three charge curves based on the two components (*p* = 2, Figure 3b) and ten components (*p* = 10, Figure 3c) using PCA. The three charge curves were selected to be the ones with the maximum capacity (curve 3), the minimum capacity (curve 1), and the median capacity (curve 2). Compared to Figure 2d, which is reconstructed based on only one expert‐extracted feature, Figure 3b shows a much more improved reconstruction accuracy for the three curves, especially for curve 1 and curve 3. The three reconstructed curves overlapped with the measured curves when the number of components is increased to ten (Figure 3c). Figure 3d further shows the average reconstruction error of all the 10066 charge curves with respect to the number of components using two different unsupervised algorithms (PCA and NMF), and the standard deviation of the errors is shown by the error bar. It needs to be noted that the NMF algorithm does not converge when the number of components is one (*p* = 1), thus it starts at *p* = 2. The result suggests that the reconstruction error decreases with the number of components in both algorithms.

### 3.3 Charge Curve Prediction Based on a Single Input Segment

**Figure 4:**
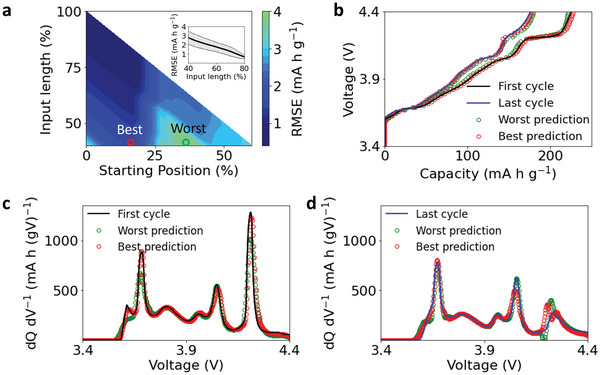
Charge curve prediction based on PCA‐extracted features. a) The average prediction error of the charge curves using the features captured by PCA. The *x* axis is the ratio between the voltage window of (*V_start_* − 3.4 *V*) and the total voltage window (4.4 *V* − 3.4 *V*), and the *y* axis is ratio between the voltage window of (*V_end_* − *V_start_*) and the total voltage window (4.4 *V* − 3.4 *V*). The inset shows the average prediction error across different starting positions with respect to the input length. b) The best and worst prediction results of the first charge curve and the last charge curve. The locations of the best and worst predictions are marked in (a). c,) The corresponding best and worst predictions of the dQ dV^−1^ curves of the two charge curves in (b).

The PCA algorithm was first applied to extract features from training charge curves to be used for predicting the entire charge curves. **Figure** **4** suggests that the model based on PCA‐extracted features can accurately predict the battery charge curve with the limited input information. A total number of 13 components (*p* = 13) was extracted to achieve a reasonable prediction accuracy on the validation set. Figure 4a shows that the prediction accuracy depends on the input data, which can be defined by the starting voltage (viz., starting position) and the voltage window (viz., length of the input data). A selected input length from 40% to 100% of the charge curve was shown to highlight the prediction accuracy. The inset shows the average prediction error with different input lengths, and the error bars represent the standard deviation of the errors across different starting positions. The result reveals that the model based on PCA‐extracted features generally has a better prediction performance when the input sequence has a starting position at 10–25%, which may depend on the investigated cathode and anode materials that determine the shape of the charge curve. The maximum relative error is 1.8% (4.0 mA h g^−1^) with 40% of input length, regardless of the starting position. The averaged relative error is only 1.3% (2.8 mA h g⁻^1^) with 40% of input, and it further reduces to less than 1.0% (2.2 mA h g⁻^1^) when the input goes beyond 50%.

The averaged prediction errors of all the test data (2013 charge curves) are further plotted in Figure S3 (Supporting Information). The large prediction error at the bottom left corner in Figure S3 (Supporting Information) is caused by the limited meaningful input information between 3.4–3.6 V, which feeds a capacity value of zero into the model. In actual battery applications, the amount of input data depends on the charge time, which is directly related to the capacity rather than the voltage. No zero capacity values will be fed into the model, and the poor prediction performance at the bottom left region can be avoided. Figure S4 (Supporting Information) shows the evolution of the averaged prediction error with respect to the input length. The averaged prediction error reaches 6.5 mA h g⁻^1^ with only 20% of input length, corresponding to 3.0% of the relative error when normalized by the maximum specific capacity of 220 mA h g⁻^1^. Moreover, the NMF‐extracted features and AE‐extracted features can also be used for predicting the entire charge curves, and the performance of the model is shown in Figures S5 and Figure S6 (Supporting Information). Both show high prediction accuracy, but slightly worse than the performance of the model based on the PCA‐extracted features. Therefore, no further analysis was conducted on these two models for the single‐segment input.

To visualize the performance of the model based on PCA‐extracted features, we show in Figure 4b the prediction of the charge curves with the largest charge capacity (equivalent to the first cycle) and 80% of the largest charge capacity (equivalent to the last cycle), where 40% of the charge curve is chosen as the input. As the prediction accuracy depends on the starting position, the starting positions of the best and worst predictions are marked in Figure 4b, which displays both the best and the worst prediction results of the charge curve in the first and the last cycle. The prediction error of the first cycle is 1.83 mA h g⁻^1^ (0.83% relative error) and 3.28 mA h g⁻^1^ (1.49% relative error) for the best and worst prediction, respectively. And the prediction error of the last cycle is 1.31 mA h g⁻^1^ (0.60%) and 2.99 mA h g⁻^1^ (1.36%) for the best and worst predictions, respectively. The small prediction errors suggest an outstanding performance of the model based on the PCA‐extracted features.

Moreover, the corresponding dQ dV^−1^ curves derived from the charge curves are shown in Figure 4c,d. The dQ dV^−1^ curve has been reported to be a versatile tool for diagnosing battery degradation mechanisms, such as loss of active materials, impedance increase, and lithium plating.^[^ ^38^ ^]^ A good match among these dQ dV^−1^ curves highlights the significance of the prediction method. Thus, a full charge curve at a constant current is no longer needed to evaluate the health status of batteries, which is time‐consuming to collect and, in certain cases, unrealistic. Instead, a partial charge curve is sufficient to construct the full dQ dV^−1^ curve for analyzing the degradation mechanisms of batteries using the methodology that we developed here.

### 3.4 Charge Curve Prediction with Multiple Separated Input Segments

**Figure 5:**
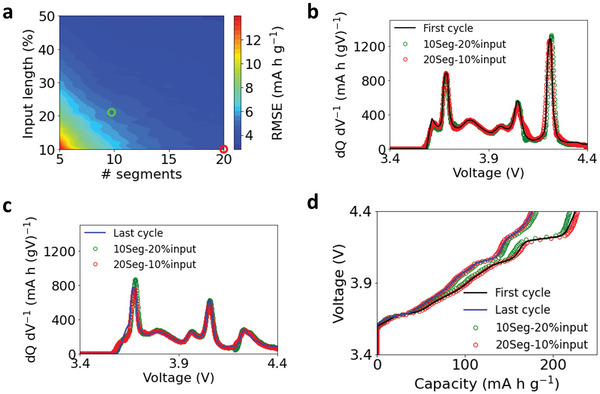
Charge curve prediction based on multiple separated input segments. a) The prediction error of the model based on AE‐extracted features. The *x* axis is the number of segments, and the *y* axis is the total length of the input sequence. b,c) Comparison between measured and predicted dQ dV^−1^ curves of b) the first cycle (with the maximum charge capacity) and c) the last cycle (80% of the maximum charge capacity). Two different types of inputs are examined, which are marked in (a). d) The corresponding charge curves calculated from the dQ dV^−1^ curves in (b) and (c).

The features extracted by the three unsupervised algorithms can also predict the entire battery charge curves using multiple separated input segments. Among the three algorithms, the AE with one hidden layer containing 20 neurons has the best prediction performance on the validation set. **Figure** **5** shows the performance of the model using multiple separated input segments based on AE‐extracted features, and Figure S7 (Supporting Information) displays the performance of the model based on PCA‐extracted features and NMF‐extracted features. The segments were randomly selected on a charge curve, and the error is averaged throughout all the test data. It needs to be noted that the best feature extraction algorithms depend on the shape of the charge curve, which is determined by the materials of the two electrodes in a battery. Thus, other feature extraction algorithms may be applied for batteries with different types of electrodes to achieve optimal prediction performance.

Figure 5a indicates that the prediction error decreases with the increase in the number of segments and the total input length in the model. The model achieves high prediction accuracy when the number of segments is more than 15, even with a small input length. For example, the prediction error can be as low as 4.1 mA h g⁻^1^ (the relative error is 1.9%) with only 10% of input length when the number of segments reaches 20, which corresponds to around 12 min of charge data collected at a C/2 rate. It should be mentioned that dQ dV^−1^ curves were predicted first when applying the multiple separated input segments, which were then used to calculate the charge curves by integrating the dQ dV^−1^ curves on voltage. We also examined the performance of the model by predicting the charge curves directly from the separated charge curve segments, but the prediction accuracy is not as good, as shown in Figure S8 (Supporting Information).

Figure 5b,c compares the tested and predicted dQ dV^−1^ curves with the maximum capacity (Figure 5b, first cycle) and 80% of the maximum capacity (Figure 5c, last cycle). Two types of inputs are selected for the model: a total input length of 20% with 10 segments and a total input length of 10% with 20 segments, which are marked in Figure 5a. Increasing the number of segments is more effective in improving the prediction accuracy than increasing the input length. For example, Figure 5a shows the prediction error of the model with 10% input and 20 segments (4.0 mA h g⁻^1^) is smaller than that with 20% input and 10 segments (5.1 mA h g⁻^1^). Figure 5b,c further shows that the peak positions and intensities of the dQ dV^−1^ curves are accurately predicted by the model that uses 20 segments and 10% of input length as the input. By comparison, there is a slight mismatch of peak intensities at 4.15 V (Figure 5b) and 3.65 V (Figure 5c) between the tested curves and the predicted ones with 10 segments and 20% of input length as the input. Such a mismatch can lead to over‐ or under‐estimation of the total charge capacity, as shown in Figure 5d.

### 3.5 Charge Curve Prediction for Different Batteries

**Figure 6:**
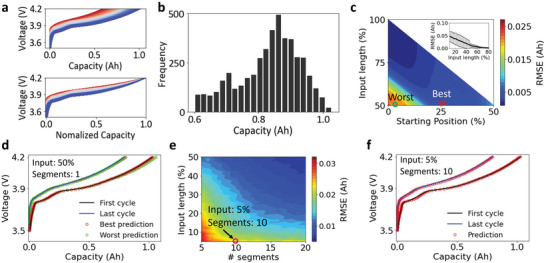
Applying the methodology to other battery chemistries. a) Charge curves and normalized charge curves of batteries taken from the Center for the CALCE dataset. b) Distribution of the charge capacity of all the 4522 charge curves. c) Performance of the model based on PCA‐extracted features and a single input segment. The inset shows the average prediction error across different starting positions. d) The best and worst prediction results of the first charge curve and last charge curve. The corresponding starting positions are marked in (c). e) Performance of the model based on the PCA‐extracted features and multiple separated input segments. f) Prediction results of the first and last charge curves based on only 5% of the input length and 10 segments. The corresponding input condition is marked in (e).

To evaluate the applicability of the methodology developed in this work, we applied the workflow to open‐access battery cycling data.^[^ ^24^ ^]^ A total number of 4522 charge curves were taken from the CALCE dataset. The CALCE dataset was tested from batteries with LiCoO_2_ as the cathode material, which is different from the LiNiO_2_ cathode that we tested in the lab, and thus shows different shapes of the charge curves, as shown in **Figure** **6**a. The accurate charge curve prediction of these batteries would illustrate the wide applicability of the developed methodology.

The batteries from the CALCE dataset show a maximum charge capacity of ≈1 Ah and a minimum capacity of 0.6 Ah. Figure 6a displays all the charge curves with the same charging rate (C/2) and the corresponding normalized charge curves. Figure 6b shows the charge capacity distribution of the 4522 curves. The decrease in the charge capacity could be attributed to the loss of active materials, growth of the resistance, and other mechanisms that can hardly be extracted or quantified by human experts. The three unsupervised learning algorithms (PCA, NMF, and AE) were applied to extract features to be fed into the multiple linear regression model for predicting the health status of the battery.

Figure 6c and Figure S9 (Supporting Information) show the performance of the model for predicting the overall charge curve using one continuous input segment. Overall, the model based on the PCA‐extracted features outperforms the model based on the other algorithms‐extracted features in the validate set, which were further used to predict the entire charge curves of the CALCE battery. Figure 6c suggests that the prediction error depends on the starting position and the length of the input data. A relatively large error appears in the bottom left corner that corresponds to 0–15% of the starting position, which is also shown in Figure S10 (Supporting Information) with a full range of the input length from 1% to 100%. This large error is caused by the sharp voltage increase between 3.5 and 3.7 V (Figure 6a). As the input length was defined by the voltage range rather than the charge capacity, the starting position at around 3.5 V would lead to much less meaningful input information compared to that started at a higher voltage. To account for this drastic increase in the voltage region, we avoid this specific region when calculating the average prediction error with respect to the input length, as shown in the inset of Figure 6c. The average prediction error is less than 0.01 Ah when the input length goes beyond 50%, which corresponds to a relative error of < 1.0% after being normalized by the maximum capacity of 1 Ah.

Figure 6d displays the prediction of the charge curves with the largest charge capacity (first cycle) and 80% of the largest charge capacity (last cycle). 50% of the charge curve was chosen as the input, and two different starting positions were selected as marked in Figure 6c to represent the best and worst performances of the model. The good agreement between the prediction curves and the tested curves indicates the outstanding performance of the model. Moreover, the corresponding dQ dV^−1^ curves derived from the charge curves also match well between the prediction and the test data (Figure S11, Supporting Information), including the positions and intensities of all the peaks.

Figure 6e shows the performance of the model with multiple separated input segments based on the PCA‐extracted features. The results show that the prediction accuracy increases with the increase in the number of segments and the input length. When the number of segments is more than 10, the prediction error is close to 0.02 Ah (relative error is 2.0%) with only 5% of the input length. Figure 6f displays the performance of the model to predict the charge curves in the first and the last cycle with 5% of the total input length and 10 separated segments. Figure S12 (Supporting Information) further displays the corresponding dQ dV^−1^ curves derived from the charge curves. The almost perfect overlap between the predicted curves and the tested curves in both Figure 6f and Figure S12 (Supporting Information) highlights the significance of the prediction method to diagnose the health status of batteries.

Last, we investigated the effect of data size and the capability of the model in predicting the maximum capacity (SOH). To evaluate the effect of data size on the performance of the developed methodology, we randomly selected 3000, 1500, and 500 charge curves from the CALCE dataset (4522 in total). Figure S13 (Supporting Information) compares the prediction accuracy of the model with different sizes of training datasets when a single segment is used as the input. The result suggests that the size of the dataset has a negligible effect on the performance of the model. It can still accurately predict the entire charge curve even if only 500 charge curves are used. Figure S14 (Supporting Information) further indicates that the size of the dataset has little effect on the performance of the model when multiple segments are used as the input. These results highlight the robustness of the developed approach in predicting the entire charge curve, which will be encouraging for practical applications where a limited dataset is available. Moreover, Figure S15 (Supporting Information) displays a parity plot of the maximum capacity. A single segment with 40% of the charge curve is used as the input to showcase the capability of SOH prediction. A less than 1% train and test error indicate the high accuracy of the developed methodology in predicting battery SOH.

### 3.6 Moving forward to the Real‐World Applications

The proposed methodology for predicting battery cycle life includes two steps: feature extraction and multiple linear regression. Different from the previous literature that treats the data‐driven method as a “black box”,^[^ ^8^ ^]^ the feature extraction step captures important information about the battery system that reflects the degradation mechanisms, such as loss of active materials, impedance growth, and increase of reaction heterogeneity. Moreover, the linear regression step provides the parameters that can be used to predict the health status of the battery, including remaining useful life, SOC, and an entire charge curve. Therefore, the developed methodology has wide application in understanding aging mechanisms, predicting health status, and providing advice to customers to optimize the application of batteries in their devices. However, there are some gaps between this study and the real‐world applications of the method in a BMS, which warrants further investigation.

First, the charge curves in the study were collected at a constant C‐rate (C/2) and at room temperature. But the current and temperature vary in real‐world battery applications. Collecting and selecting the appropriate information to be used as the input will be an important step to improve the robustness of the model. An alternative solution is to develop a more robust model that can take all the information (current, temperature, voltage, capacity, etc.) as the inputs, and neural networks could be a candidate for solving the problem.

Second, batteries with different types of chemistries (cathodes and anodes) have been widely used, which leads to different shapes of cycling curves. Although we examined two different batteries and demonstrated the applicability of the methodology in both cases, further evaluation of the model in other battery systems is needed. Moreover, the optimal feature extraction algorithms may differ from one battery system to another. More algorithms should be examined to obtain the model with the best prediction performance for a specific battery system.

Moreover, it is worth noting that dQ dV^−1^ curves are generally plotted at low rates (C/10 or below) to investigate the thermodynamical aspects of the battery. The accuracy of the dQ dV^−1^ analysis also depends on the quality of the measurement data.^[^ ^38^ ^]^ For example, it is important to ensure environmental consistency during the test (temperatures and contacts), and the sampling rate should be reasonable to ensure enough data points for analysis and avoid large data files. Best practices for testing have been introduced in the literature.^[^ ^39^ ^]^ In our study, the charging rate was C/2 for the batteries. The prediction accuracy of the dQ dV^−1^ curves is expected to increase with a slower charging rate, which warrants further investigation.

Finally, correlating the algorithms‐extracted features with the degradation mechanisms of a battery is an important step to deepen our understanding of the system. As a battery is a complex nonlinear system, the evolution of the electrodes (cathode and anode), electrolytes, and the interface between them could lead to a change in the capacity and resistance, which will be reflected in the cycling curve. Uncovering the battery degradation mechanisms and quantifying their effect on the shape of the charge curve could help build physics‐informed models to reach an optimal prediction of battery performance.

## 4 Conclusion

Data‐driven methods have a superior ability to capture features in cycling curves. The features can be correlated to the aging mechanism of batteries, such as loss of active materials and growth of resistance. Moreover, these features can be combined with a multiple linear regression model to predict a complete cycling curve based on a limited portion of it. We demonstrate that a single continuous segment and multiple separated segments can be used as the input to predict the complete cycling curve. The model achieves a 2% prediction error of an entire charge curve using only 10% of the curve as the input for the LiNiO_2_‐based batteries and achieves the same accuracy with only 5% of the curve as the input for the LiCoO_2_‐based batteries. The complete charge curve can be used to evaluate the health status of batteries, which can not only guide the use of batteries but prevent accidents and malfunctions.

## Conflict of Interest

The corresponding author (A. M.) is a co‐founder of TexPower, Inc., a start‐up company focusing on cobalt‐free cathode materials for lithium‐based batteries.

## Supporting information

Supporting Information

## Data Availability

The data that support the findings of this study are available from the corresponding author upon reasonable request.
