# Supplementary material for: Battery Charge Curve Prediction via Feature Extraction and Supervised Machine Learning
Source: Adv Sci (Weinh). 2023 Jul 2;10(26):2301737. doi: 10.1002/advs.202301737 (PMC10502833; doi:10.1002/advs.202301737)
Supplement: Supplementary file 1 — Supporting Information [file ADVS-10-2301737-s001.pdf]

ADVANCED SCIENCE
Open Access

Supporting Information

for *Adv. Sci.*, DOI 10.1002/advs.202301737

Battery Charge Curve Prediction via Feature Extraction and Supervised Machine Learning

*Laisuo Su, Shuyan Zhang, Alan J. H. McGaughey, B. Reeja-Jayan and Arumugam Manthiram**

WILEY-VCH

Supporting Information

Battery Charge Curve Prediction via Feature Extraction and Supervised Machine Learning

*Laisuo Su, Shuyan Zhang, Alan J. H. McGaughey, B. Reeja-Jayan, Arumugam Manthiram**

Dr. L. Su, Dr. A. Manthiram

Materials Science and Engineering Program & Texas Materials Institute, The University of Texas at Austin, Austin, TX 78712-1591, United States

Email: manth@austin.utexas.edu

Dr. S. Zhang, Dr. A. McGaughey, Dr. B. Jayan

Department of Mechanical Engineering, Carnegie Mellon University, Pittsburgh, Pennsylvania 15213, United States

*Corresponding author. Tel: +1-512-471-1791; fax: +1-512-471-7681.

E-mail address: manth@austin.utexas.edu (A. Manthiram)

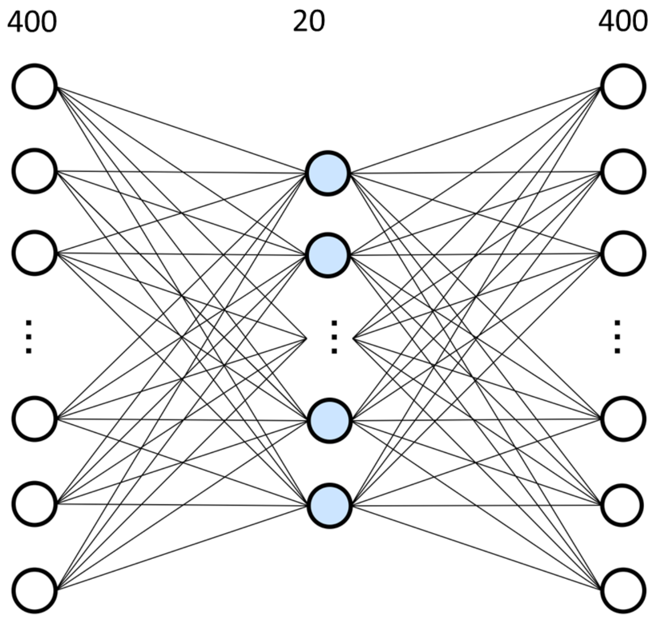

Fig. S1 Structure of the autoencoder to extract hidden features from battery charge curves.

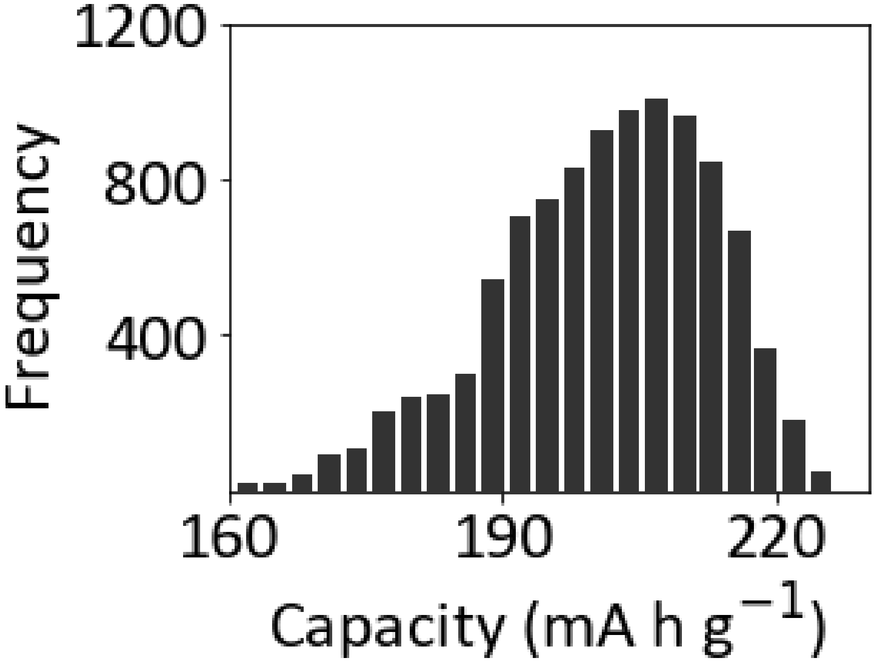

Fig. S2 Distribution of the charged capacity of the 10,066 curves used in this study.

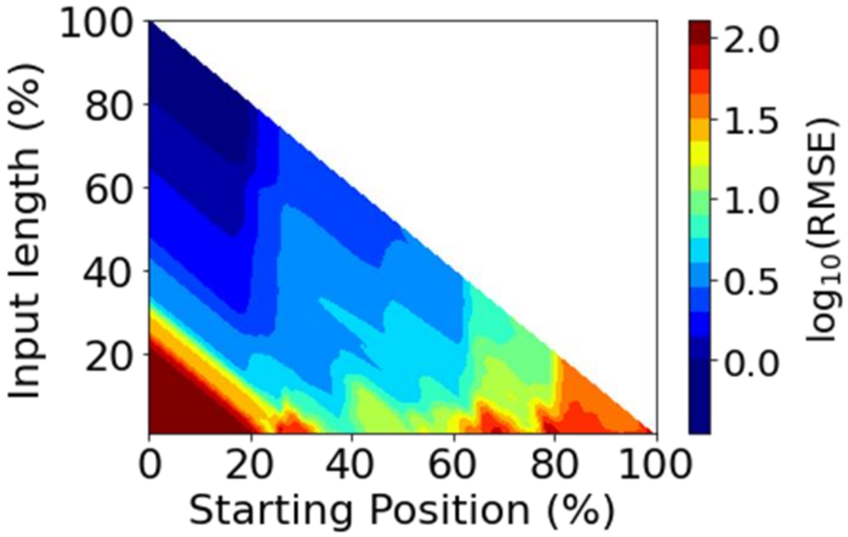

Fig. S3 The averaged prediction error of the charge curves using the features captured by PCA. A full range of the input length from 1% to 100% of the charge curve is shown.

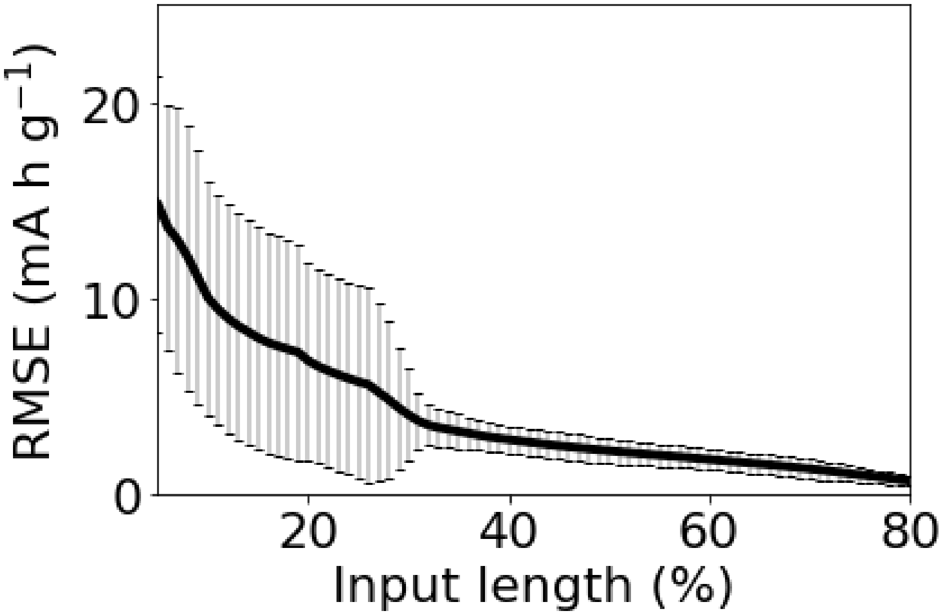

Fig. S4 The averaged prediction errors with respect to the length of the input using the model based on PCA-extracted features. The averaged values and standard deviations are calculated across different starting positions. The bottom left region with large prediction errors is omitted when calculating the average errors.

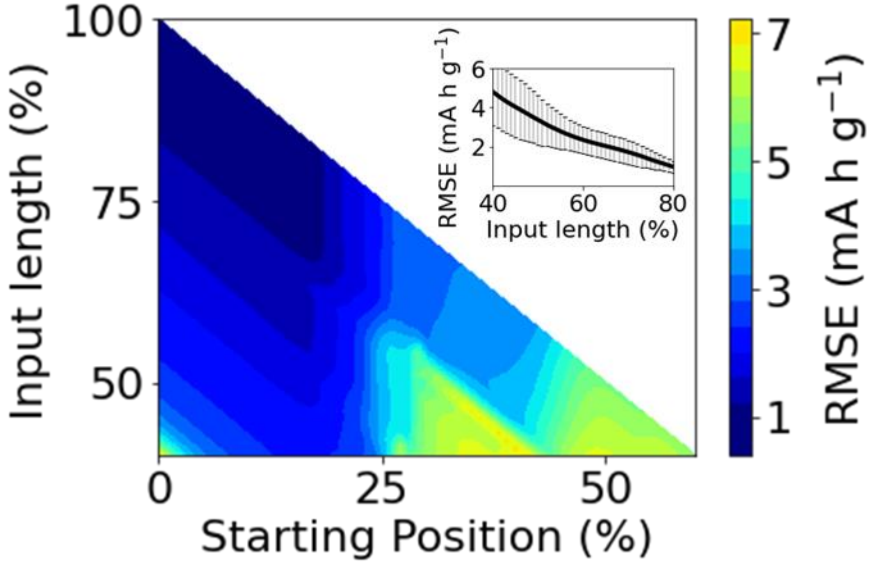

Fig. S5 The averaged prediction error of the charge curves using the features captured by NMF. The inset shows the average prediction error across different starting positions with respect to the length of the input.

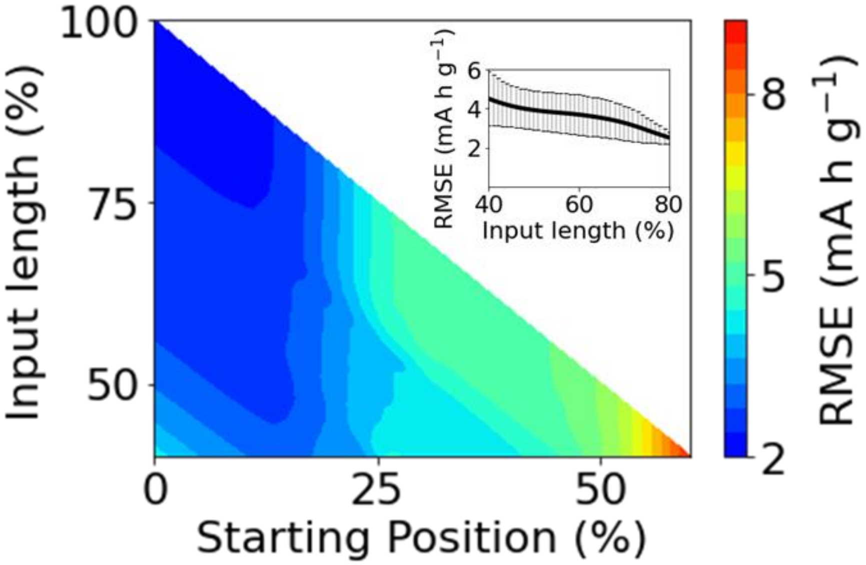

Fig. S6 The averaged prediction error of the charge curves using the features captured by AE. The inset shows the average prediction error across different starting positions with respect to the length of the input.

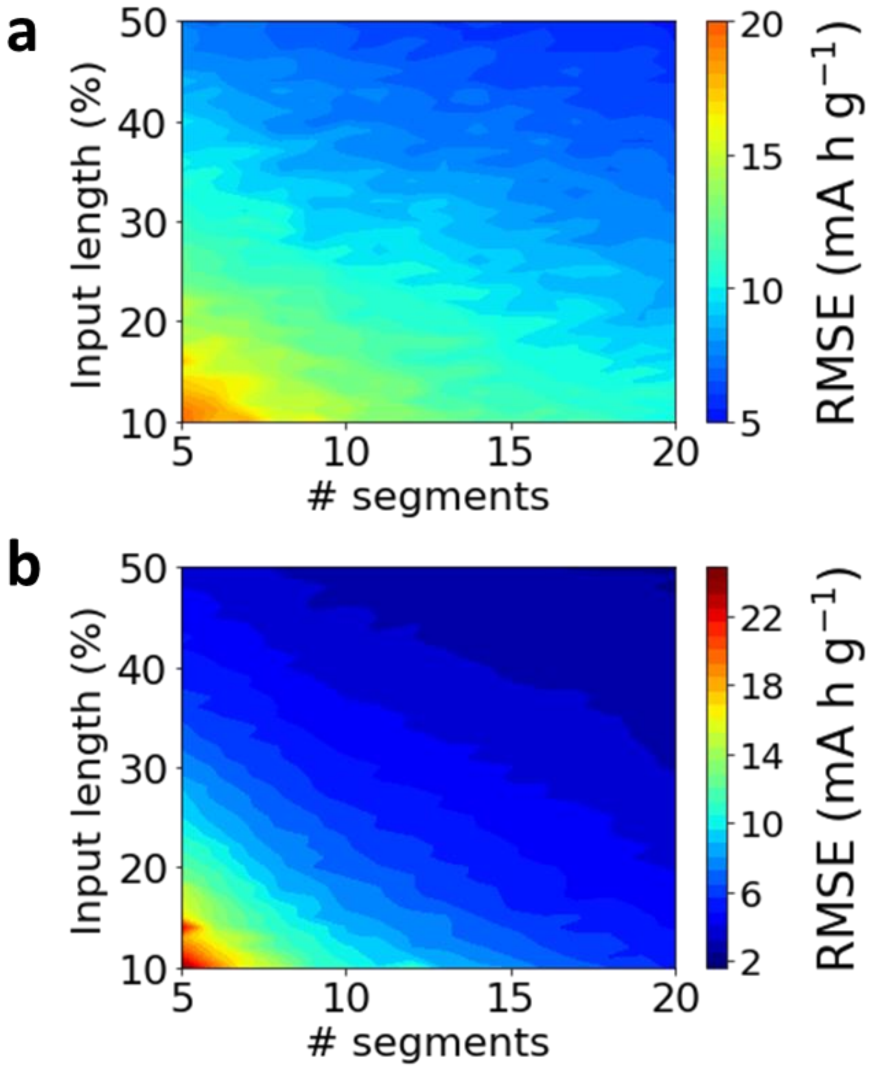

Fig. S7 Prediction accuracy of the model based on (a) PCA-extracted features and (b) NMF-extracted features. Multiple separated segments are used as the input of the model.

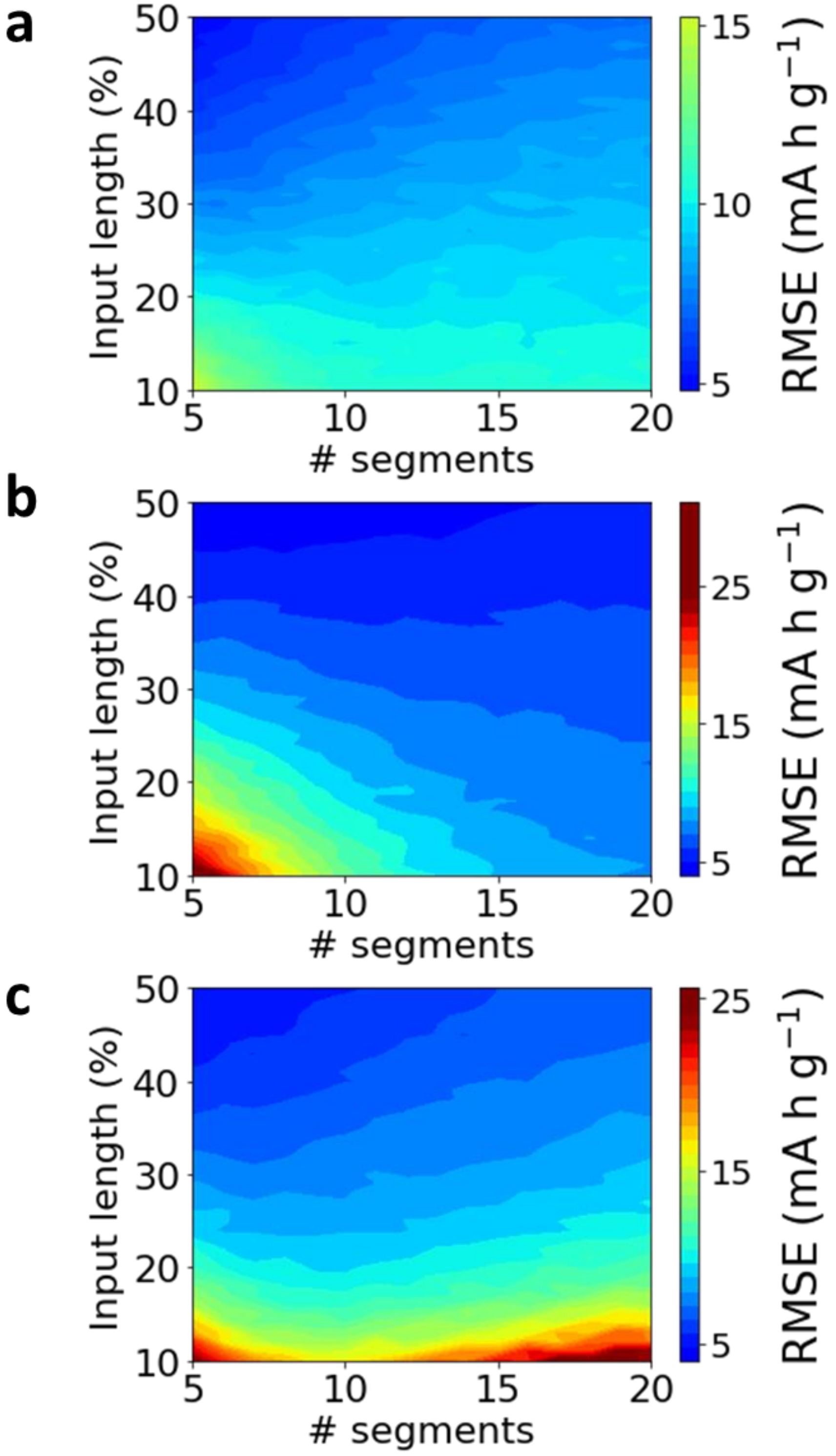

Fig. S8 Charge curve prediction based on multiple separated segments of the charge curve. The average prediction error of the charge curves based on (a) PCA-extracted features, (b) NMF-extracted features, and (c) AE-extracted features.

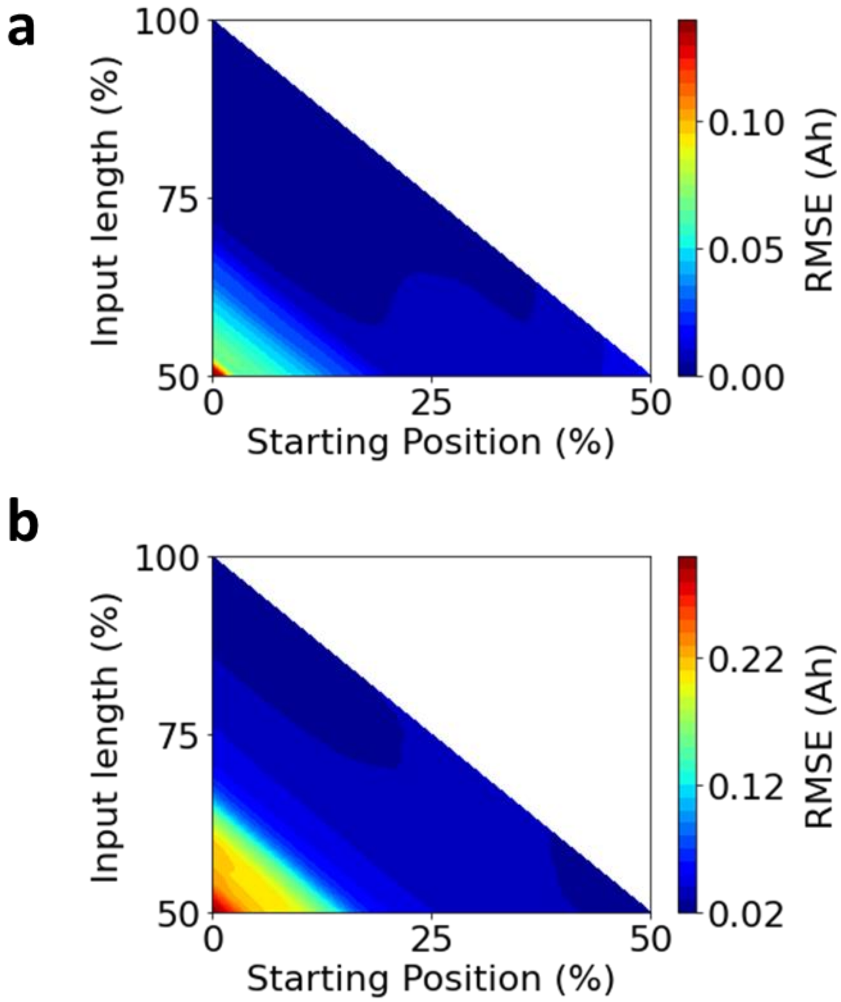

Fig. S9 Performance of the model to predict the CALCE battery charge curves based on (a) NMF-extracted features and (b) AE-extracted features. The rrange of the input length from 50% to 100% of the charge curve is selected to highlight the accuracy of the model.

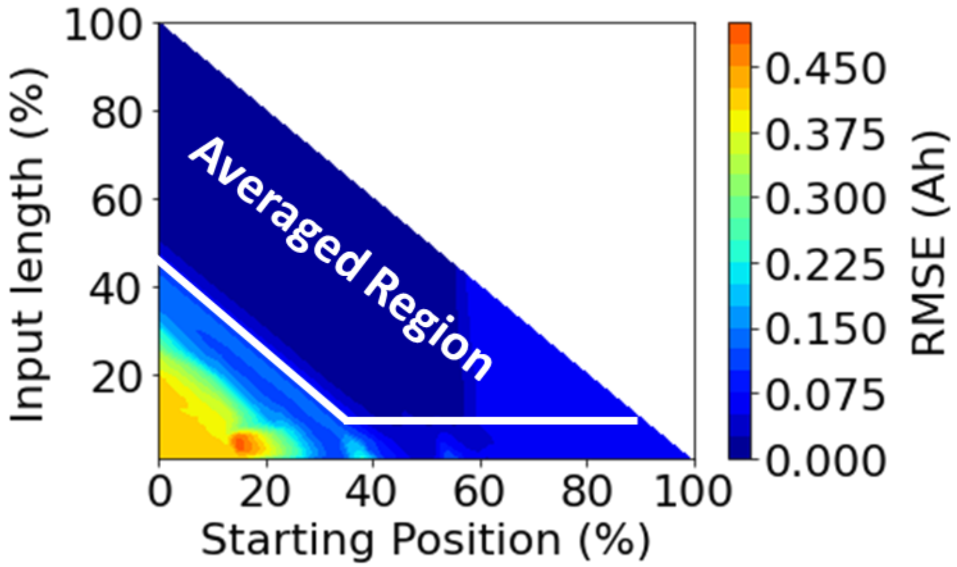

Fig. S10 Selected region for calculating the average prediction error of the model using the PCA-extracted features in CALCE data.

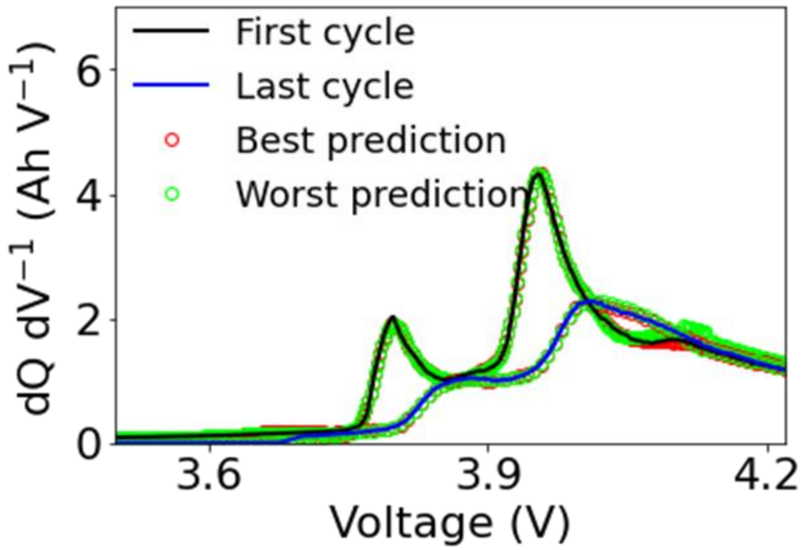

Fig. S11 The best and worst prediction of dQ dV^{-1} curves of the two charge curves of the CALCE data, which has the maximum charge capacity (first cycle) and the 80% of the maximum charge capacity (last cycle), respectively.

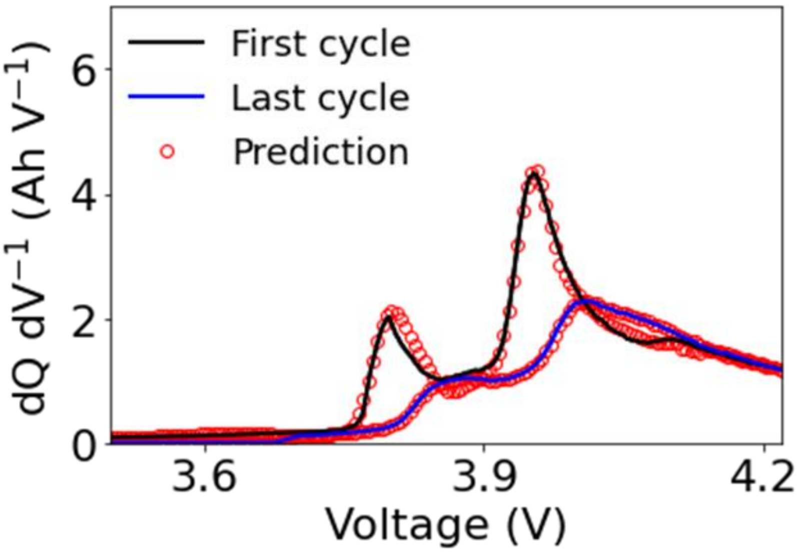

Fig. S12 Prediction of dQ dV^{-1} curves of the two charge curves of the CALCE data based on the multiple separated input segments. The total input length is 5%, and the number of segments is 10.

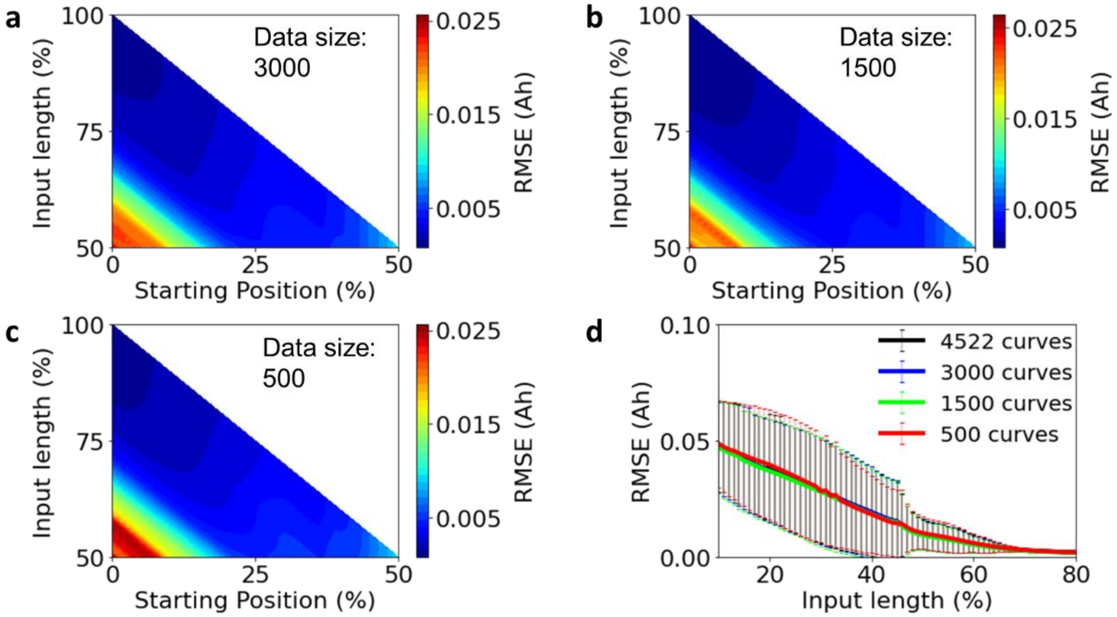

Fig. S13 Effect of data size on the performance of the model when a single segment is used as the input. The total numbers of charge curves are (a) 3000, (b) 1500, and (c) 500. (d) A comparison of the average RMSE across all starting positions in (a) - (c). The CALCE dataset is selected here for the investigation.

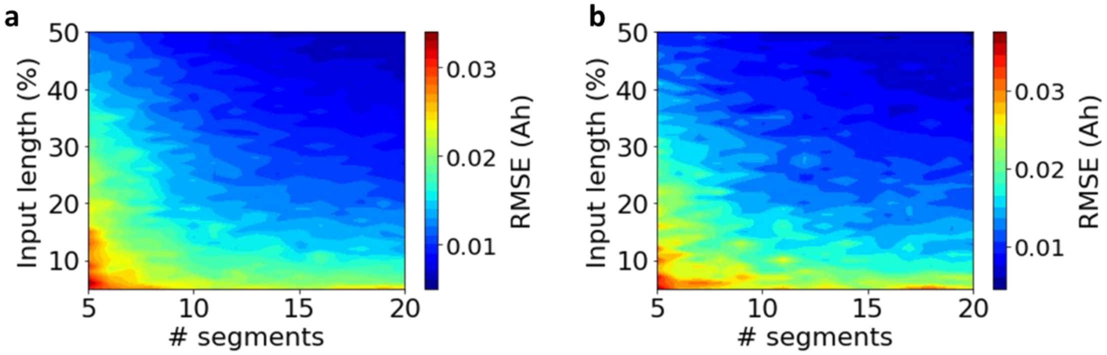

Fig. S14 Effect of data size on the performance of the model when multiple segments are used as the input. The total numbers of charge curves are (a) 1500 and (b) 500.

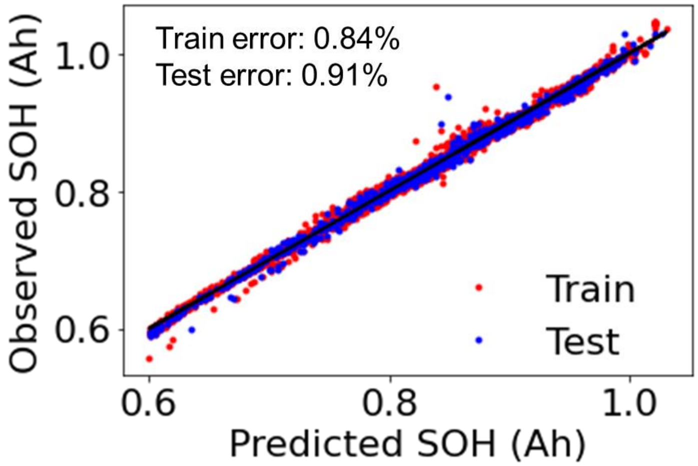

Fig. S15 A parity plot to show the performance of the model in predicting the SOH of batteries. The input is a single segment with 40% of the charge curve.
